# Hypercalciuria following ceftriaxone a fact or myth

**DOI:** 10.12861/jrip.2015.20

**Published:** 2015-09-01

**Authors:** Anoush Azarfar, Mohammad Esmaeeli, Yalda Ravanshad, Sepideh Bagheri, Ezzat Khodashenas, Fatemeh Ghane-Sharbaf, Majid Malaki, Amir-Hossein Mohamadian

**Affiliations:** ^1^Department of Pediatric Nephrology, School of Medicine, Mashhad University of Medical Sciences, Mashhad, Iran; ^2^Clinical Research Development Center, Ghaem Hospital, School of Medicine, Mashhad University of Medical Sciences, Mashhad, Iran; ^3^Pediatric Health Research Center, Tabriz University of Medical Sciences, Tabriz, Iran

**Keywords:** Ceftriaxone, Hypercalciuria, Children

## Abstract

**Introduction:** Nephrolithiasis is a common worldwide problem both in children and adults. Ceftriaxone as a widely used antibiotic can contribute to the formation of renal stones and hypercalciuria.

**Objectives:** To find the effect of ceftriaxone, a widely used antibiotic, on urinary calcium excretion rate in children.

**Patients and Methods:** 84 infants and children over 3 months admitted to hospital for non-renal problems. They were all previously healthy children affected with a condition mandating hospitalisation. They were randomly divided into 2 groups; those who received ceftriaxone according to their physician decision as the case group and those who did not receive antibiotics as the control group. The patients urinary calcium excretion was determined as calcium to creatinine ratio in a random urine sample in the first and third day of their admission. All data was expressed by mean ± SD and analysed by t independent and chi-square tests by SPSS 16. P* P* value less than 0.05 was significant.

**Results:** Eighty-four cases were analysed. Calcium excretion in received and non-received ceftriaxone groups was 0.13 ± 0.06 and 0.14 ± 0.02 respectively at first day of admission (* P* = 0.1). After 3 days, the urine calcium to creatinine ratio increased to 0.27 ± 0.2 and 0.26 ± 0.08 in received and non- received ceftriaxone groups (* P* = 0.8).

**Conclusion:** In children, urinary calcium excretion increases 2 times in average in a short time after admission because of gastroenteritis, and ceftriaxone is not different to other antibiotics for increase urinary calcium excretion in 3 days after admission.

**Implication for health policy/practice/research/medical education:**

While ceftriaxone is a widely used antibiotic, we aimed to test whether it could contribute to the formation of renal calculi. Results of the current study showed that urinary calcium excretion does not significantly rise after ceftriaxone administration.

## Introduction

Nephrolithiasis is a common worldwide problem both in children and adults (1). Ceftriaxone as an anion can bind to calcium and make nucleolus of bile stones (2). Other studies show pseudolithiasis occurs in 25% to 50% of children treated by ceftriaxone (3). Rising urine calcium excretion has been suggested by some researchers (4-6). The effect of ceftriaxone on urine calcium excretion was also confirmed in children who received parental ceftriaxone in (5) and adults group in both research studies and case report (4,7). In one study, nephrolithiasis occurs in 8% of patients who received ceftriaxone in spite of that normal urinary calcium to creatinine excretion (6) while in other study in Iran, nephrolithiasis may occur in 4% of children who received ceftriaxone and be cleared up to 3 months after drug cession (8). These evidences show that ceftriaxone may have different effects on calcium excretion and renal stone formation.

## Objectives

In this study, we aimed to investigate the effect of ceftriaxone on calcium excretion, and our results can help physicians to manage patients more efficiently.

## Patients and Methods

Children admitted to hospital because of different reasons who received ceftriaxone with standard dosage (1) as the case group and children who are needed to use other antibiotic except ceftriaxone considered as the control group.

### Exclusion criteria

Children with a history of renal diseases and nephrolithiasis and under treatment of nephrotoxic or drugs which have effect on calcium excretion were excluded.

### Study population

All new admitted patients who needed ceftriaxone prescription entered to the study and their urine for calcium to creatinine excretion in a random sample was taken in the first and third day after ceftriaxone prescription, in both groups of case who are under ceftriaxone therapy and control group who were treated by other antibiotic other than ceftriaxone. Hypercalciura was defined as randomized urine calcium to creatinine ratio more than 0.8 in the age under 8 months age and 0.21 for children over age 8 months. All children serum electrolyte included sodium, potassium, calcium, and urine calcium to creation in random type were measured in the first day and third day after admission.

### Ethical issues

1) The research followed the tenets of the Declaration of Helsinki; 2) informed consent was obtained, and they were free to leave the study at any time and 3) the research was approved by the ethical committee of Mashhad University of Medical Sciences.

### Statistical analysis

All data is expressed by crude number and percent, comparison of mean of results that are included of serum electrolytes, calcium to creatinine ratio between children who received ceftriaxone and non-received group were analysed by* t* independent test and the comparison of changes of urinary calcium excretion average values in first and third day after admission in both groups with and without ceftriaxone will be performed by paired *t* test and chi-square test. All analysis were performed by SPSS 16, and *P* value less than 0.05 was considered as significant.

## Results

Eighty-four children with mean age of 36 months (minimum 4 and maximum 133 months) admitted to study including 38 girls and 46 boys. They were divided into group 1 who received ceftriaxone and group 2 who did not receive ceftriaxone.

Serum creatinine did not differ between group 1 and group 2 (0.763 ± 0.33 vs 0.66 ± 0.20; *P *= 0.1). Other electrolytes such as calcium, sodium, and potassium were not different between 2 groups (*P *> 0.5; Table 1). Urinary calcium excretion based on urine calcium to creatinine excretion was 0.13 ± 0.04 in all cases without considering their group. These measures in both groups show that there is not any difference between them: 0.14 ± 0.02 in received and 0.13 ± 0.06 in not received group (*P *> 0.5). Changes of calcium excretion 3 days after admission in both groups were significant. The mean ± SD of calcium excretion was 0.26 ± 0.08 in received ceftriaxone and 0.27 ± 0.2 in not- received group, therefore there was a significant increase of calcium excretion in both groups related to first day samples (*P* < 0.0001). There was no difference in urine calcium excretion quantity between 2 groups of received and non-received ceftriaxone in the third day (*P *= 0.82; Table 1). The incidence of hypercalciuria occurrence in the third day was more common in received ceftriaxone group than the non-received group, however this difference was not significant (44% vs 28%; *P *= 0.1). Serum creatinine and electrolytes such as serum calcium, potassium, and sodium was checked in both groups which was showed there is no difference of these serum parameters in these groups during the period of surveillance on the third day.

**Table 1:** Comparison of serum and urine laboratory indices in children treated with and without ceftriaxone

**Lab tests**	**Received**	**Non-received**	***P*** ** value**
	**Mean ± SD**	**Mean ± SD**	
Serum Cr (mg/dl)	0.763 ± 0.33	0.66 ± 0.20	0.104
Serum Ca (mg/dl)	8.98 ± 0.71	8.55 ± 1.01	0.34
Serum Na (mg/dl)	138.92 ± 7.08	140.45 ± 7.58	0.35
Serum K (mg/dl)	4.12 ± 0.82	4.23 ± 0.86	0.54
Urine Ca/Cr first day	0.13 ± 0.06	0.14 ± 0.02	0.5
Urine Ca/Cr thirdday	0.26 ± 0.08	0.27 ± 0.2	0.8

## Discussion

Ceftriaxone is an antibiotic which has widely been used in both children and adult. Ceftriaxone in high concentration can bind to calcium and form stone nidus bladder stone (2), which can play an important role in forming renal stone with similar mechanism (7-10). Increase of calcium excretion is another suggested debatable mechanism for renal stone formation due to ceftriaxone usage (4-6). Avci et al (6) estimated 8.7% incidence of renal stone in someone who received ceftriaxone but there is not any change in calcium excretion rate after ceftriaxone use, while Kimata et al (5) found that there is an increase in urine calcium excretion 4 days after ceftriaxone prescription without regarding ceftriaxone dosage. There are other studies that compare the prescription of ceftriaxone versus other antibiotic types on the urine calcium excretion or stone formation. Otunctemur et al (4) found that the type of antibiotic in adult groups can play a role in increase of calcium excretion, as ceftriaxone and cephalothin raise calcium excretion in 5 days, while ampicillin does not have such a property.

Our study was performed in selective children who were hospitalized for gastroenteritis, and they were divided into 2 groups randomly. The first group received a usual dose of ceftriaxone between 50 and 75 mg/kg per day, and the second group received other types of antibiotics. Our finding show that hypercalciuria is a common finding in children during first days of admission in hospital due to gastroenteritis. The incidence of hypercalciuria was 30%, while the incidence of hypercalciuria in general population varied between 2.9% and 3.8%, which may cause many complaints such as dysuria, hematuria, urgency, urine infection and renal stone (11-13). In fact, our study shows that the incidence of hypercalciuria in ill children increases 10 times in first days of their diseases. This finding is similar to other studies that estimate hypercalciuria to be between 45% and 54% after seventh day of admission without regarding the patients’ antibiotic type usage (14). In this study, we focus on the role of antibiotic types specially ceftriaxone to investigate if it causes an increase of calcium excretion in children with gastroenteritis, but our findings show that hypercalciuria increased 2 times in both patient who received or did not receive ceftriaxone after the third day.

This study show hypercalciuria is a common finding in children admitted to hospital and ceftriaxone solely cannot play a major role for stone formation only by increasing urinary calcium excretion and other factors like as immobilization, dehydration and unknown factors should be considered as major factors that may have role in renal stone formation in children following use of ceftriaxone.

## Conclusion

After 3 days of hospitalization, calcium excretion increase in urine, probably due to multifactorial factor and ceftriaxone is not different from other antibiotics for calcium excretion rate in children age group.

## Limitations of the study

Results of this study may be rather limited due to its small sample size. Further studies with larger populations is suggested to better detect this aspect of antibiotic therapy in children.

## Authors’ contribution

AA and YR: study design, preparation of manuscript, and final revision. ME: study design, manuscript edition, and final revision. SB and EK: data gathering, data interpretation, and manuscript preparation. FGS and MM: data interpretation, manuscript preparation, and final revision. AHM: data gathering and manuscript preparation.

## Conflicts of interest

The authors declared no competing interests.

## Ethical considerations

Ethical issues (including plagiarism, misconduct, data fabrication, falsification, double publication or submission, redundancy) have been completely observed by the authors.

## Funding/Support

This study is extracted from the thesis of Amir-Hossein Mohammadian (Research project No. 910430).
